# Metabolic Syndrome Is Associated With Poor Prognosis in Patients With Breast Cancer Receiving Neoadjuvant Therapy

**DOI:** 10.1002/cam4.70484

**Published:** 2024-12-20

**Authors:** Youzhao Ma, Jingyang Zhang, Dechuang Jiao, Xiuchun Chen, Zhenzhen Liu

**Affiliations:** ^1^ Department of Breast Disease The Affiliated Cancer Hospital of Zhengzhou University & Henan Cancer Hospital Zhengzhou China

**Keywords:** breast cancer, disease‐free survival, metabolic syndrome, overall survival, pathological complete response

## Abstract

**Purpose:**

Few studies with a large sample size are available on patients with metabolic syndrome (MetS) receiving neoadjuvant treatment (NAT) for breast cancer. This study aimed to investigate the impact of MetS on the prognosis of patients with breast cancer undergoing NAT.

**Methods:**

The data of patients with breast cancer receiving NAT at our center from January 2017 to December 2019 were retrospectively analyzed. A chi‐square test and logistic regression model were applied to ascertain the factors associated with MetS and pathological complete response (pCR). The Cox proportional risk model was employed for univariate and multivariate analyses of disease‐free survival (DFS) and overall survival (OS).

**Results:**

Of the 910 patients enrolled, 164 (18.0%) were diagnosed with MetS, 568 (62.4%) with stage II, and 342 (37.6%) with stage III. Postmenopausal status (*p* = 0.045) and stage III (*p* = 0.009) were associated with a higher incidence rate of MetS. MetS was associated with a lower pCR rate (*p* = 0.027). The 5‐year DFS (83.7% vs. 73.1%, *p* = 0.001) and OS (92.8% vs. 85.5%, *p* = 0.001) of the non‐MetS group were significantly better than those of the MetS group. In premenopausal women, the DFS (*p* = 0.001) and OS (*p* = 0.025) of the non‐MetS group were significantly better than those of the MetS group. No significant differences were noted in DFS (*p* = 0.270) or OS (*p* = 0.078) between the two groups in postmenopausal women. In the Cox proportional risk model, MetS acted as an independent factor associated with DFS (HR = 1.705, 95% CI: 1.201–2.421, *p* = 0.003) and OS (HR = 1.874, 95% CI: 1.149–3.055, *p* = 0.012).

**Conclusion:**

MetS was associated with poor prognosis in patients with breast cancer receiving NAT. Hence, close attention should be paid to patients with breast cancer who have MetS.

**Abbreviations**
95% CI: 95% confidence interval
BCSS: breast cancer‐specific survival
DFS: disease‐free survival
DM: diabetes mellitus
ER: estrogen receptor
FISH: fluorescence in situ hybridization
HER2: human epidermal growth factor receptor 2
HR: hormone receptor
IHC: immunohistochemistry
MetS: metabolic syndrome
NAT: neoadjuvant therapy
OS: overall survival
pCR: pathological complete response
PR: progesterone receptor

## 1 Introduction

Globally, there are approximately 2.3 million new cases of female breast cancer and approximately 670,000 deaths annually [1], which seriously threatens the life and health of women. Neoadjuvant therapy (NAT) is a systemic treatment for early breast cancer before radical surgery. For breast cancer with a locally advanced stage or highly invasive tumors, NAT can not only achieve the goal of downstaging cancer surgery or breast‐conserving surgery but also predict the prognosis according to the efficacy and guide the development of postoperative therapeutic regimens [2].

Metabolic syndrome (MetS) refers to the pathological state, in which disorders related to the metabolism of proteins, fats, carbohydrates, and other substances occur, and it highlights the potential increased risk of diseases [3, 4]. MetS is defined by a set of related factors, including elevated blood pressure and blood sugar, abdominal obesity, decreased high‐density lipoprotein cholesterol (HDL‐C) content, and increased triglyceride content [5, 6, 7]. In the Western population, the incidence of MetS increases significantly with age, resulting in a prevalence rate of 40%–45% in individuals aged ≥ 50 years [8, 9]. A previous study has confirmed that elderly and postmenopausal women are more likely to develop MetS [6]. Owing to societal aging, MetS has become a global public health issue that is receiving increasing attention.

The components of MetS are believed to be related to the occurrence and development of cancer [10]. MetS has been shown to be associated with an increased risk of developing multiple cancers [11] and is significantly associated with poor prognosis [12]. Studies have reported that MetS is associated with the adverse pathological characteristics of breast cancer [13] and the increased risk of women suffering from breast cancer [11, 14, 15, 16]. Retrospective studies have observed that MetS is a poor prognostic factor for breast cancer [17, 18, 19, 20]. In addition, a prospective study found that patients with breast cancer and MetS have poor overall survival (OS) and breast cancer‐specific survival (BCSS) [6].

Nonetheless, the effect of MetS on the treatment efficacy and prognosis of patients with breast cancer undergoing NAT lacks data validation of large‐sample studies. Hence, this study aimed to evaluate the association between MetS and clinicopathological characteristics and to analyze the impact of MetS on pathological complete response (pCR) after NAT and on the prognosis of breast cancer.

## 2 Materials and Methods

### 2.1 Patient Selection

In this study, the clinicopathological characteristics of patients with breast cancer who received NAT, followed by surgery at Henan Cancer Hospital from January 2017 to December 2019, were retrospectively collected and analyzed. This research was conducted in accordance with the tenets of the Helsinki Declaration and was approved by the Medical Ethics Committee of Henan Cancer Hospital (Approval Number: 2022–299). Patients were included based on the following criteria: (1) women; (2) stage II–III breast cancer (based on the seventh edition of the TNM staging system of the American Joint Committee on Cancer); (3) received NAT (chemotherapy ± targeted therapy); (4) data available on the expression of human epidermal growth factor receptor 2 (HER2), estrogen receptor (ER), progesterone receptor (PR), and Ki‐67 index before NAT; (5) underwent curative surgery after NAT, and postoperative pathological data were complete; and (6) follow‐up data available. The exclusion criteria were as follows: (1) metastatic breast cancer; (2) bilateral breast cancer; (3) inflammatory breast cancer; (4) breast cancer with other primary malignant tumors; and (5) suffering from other diseases that seriously affect body mass index (BMI), blood pressure, blood sugar, or lipid metabolism.

### 2.2 Data Collection

Data on clinical features (including age at diagnosis, height, and weight before NAT, menopausal status, clinical TNM stage, pre‐NAT blood pressure, fasting blood glucose, triglycerides, and HDL‐C), pathological features (ER, PR, HER2, and Ki‐67 index before NAT), NAT regimens (including chemotherapy and/or anti‐HER2 targeted therapy), type of surgery, radiotherapy, pCR, and follow‐up data (date of recurrence or death) were collected. The parameters of fasting blood glucose, triglycerides, and HDL‐C were tested uniformly in the biochemical laboratory of Henan Cancer Hospital. Information on ER, PR, HER2, and Ki‐67 was obtained from pathological reports of biopsy specimens of primary breast tumors before NAT.

Both ER and PR were defined as negative if < 1% and positive if ≥ 1%. Hormone receptor (HR) negativity was defined as ER < 1% and PR < 1%. In addition, HR positivity was defined as ER/PR ≥ 1%. HER2 expression was detected using immunohistochemical (IHC) analysis combined with fluorescence in situ hybridization (FISH) according to the HER2 testing guidelines of the American Society of Clinical Oncology/College of American Pathologists. IHC 0, 1+, and 2+ and FISH without HER2 gene amplification were defined as HER2‐negative; IHC 1+, 2+, and FISH− as HER2 low; and IHC 3+, 2+, and FISH+ as HER2‐positive. As per the National Cholesterol Education Program Adult Treatment Panel III standard [5], those who met three of the following five criteria were diagnosed with MetS: (1) BMI ≥ 25 kg/m^2^; (2) blood pressure ≥ 130/85 mmHg or a history of hypertension; (3) fasting blood glucose ≥ 6.1 mmol/L, blood glucose ≥ 7.8 mmol/L at 2 h after a meal or a history of diabetes mellitus (DM); (4) triglycerides ≥ 1.7 mmol/L; and (5) HDL‐C < 0.9 mmol/L. Owing to the lack of waist circumference data, BMI was used [21]. PCR was defined as the absence of residual invasive cancer (ypT0ypN0M0/ypTisypN0M0) in samples from the breast and axillary lymph nodes.

### 2.3 Follow‐Up

All patients were regularly followed up, and the findings were recorded by professionals. Disease‐free survival (DFS) was defined as the time period from surgery to disease recurrence, death from any cause, or the last follow‐up. OS was defined as the time period from diagnosis to death from any cause or the last follow‐up.

### 2.4 Statistical Analysis

SPSS 23.0 software was used for statistical analysis. Descriptive statistics were utilized to determine the frequency and percentage of variables and the median of continuous variables. For baseline feature comparison, an independent sample's *t*‐test or nonparametric test was used for continuous variables, and a chi‐square test or Fisher's exact test was used for categorical variables. Furthermore, the chi‐square test and logistic regression model were used to ascertain the factors that influenced MetS and pCR. In addition, the Cox proportional risk model was used for univariate and multivariate analyses of DFS and OS. Those variables with *p* < 0.05 in univariate analysis were included in multivariate analysis, and finally, independent risk factors affecting the prognosis of breast cancer were obtained. Bilateral *p*‐values < 0.05 were considered statistically significant. The Kaplan–Meier survival curve was plotted to assess the impact of risk factors on prognosis. The log‐rank test was applied to compare the survival curves of two or more groups.

## 3 Results

### 3.1 Patient Characteristics According to MetS Status

The median age at diagnosis for the 910 patients included in this study was 49 years. There were 164 (18.0%) patients in the MetS group and 746 (82.0%) in the non‐MetS group. There were 574 (63.1%) premenopausal and 336 (36.9%) postmenopausal women. Of the enrolled patients, 361 (39.7%) were HR‐negative and 549 (60.3%) were HR‐positive. In addition, there were 312 (34.3%) patients with the luminal (HER2‐negative) subtype (HR+/HER2−), 227 (24.9%) with the luminal (HER2‐positive) subtype (HR+/HER2+), 138 (15.2%) with the HER2‐positive subtype (HR–/HER2+), and 233 (25.6%) with the triple‐negative (HR–/HER2–) subtype. A total of 51 patients received taxane‐based chemotherapy, 221 received taxane‐based chemotherapy + anti‐HER2 targeted therapy, 571 received anthracycline‐based chemotherapy, and 67 received anthracycline‐based chemotherapy + anti‐HER2 targeted therapy. The results of the univariate analysis indicated that MetS was associated with age at diagnosis (*p* < 0.001), menopausal status (*p* < 0.001), and cTNM staging (*p* = 0.004). After multivariate analysis, menopausal status and cTNM staging were associated with the incidence of MetS. Compared with premenopausal women, postmenopausal women exhibited a higher incidence rate of MetS (OR = 1.630, 95% confidence interval [95% CI]: 1.012–2.628, *p* = 0.045). Moreover, the incidence of MetS in patients with stage III was higher than that in patients with stage II disease (OR = 1.581, 95% CI: 1.120–2.232, *p* = 0.009) (Table 1).

**TABLE 1:** Clinicopathological characteristics associated with MetS.

Characteristics	Total	Non‐MetS group	MetS group	Univariate analysis		Multivariate analysis		
		*N* (%)	*N* (%)	*χ* ^2^	*p*	OR	95% CI	*p*
Age at diagnosis		746 (82.0)	164 (18.0)					
≤ 50	501	431 (57.8)	70 (42.7)	12.375	< 0.001	Ref		
> 50	409	315 (42.2)	94 (57.3)			1.280	0.793–2.065	0.312
Menopausal status								
Premenopausal	574	493 (66.1)	81 (49.4)	16.091	< 0.001	Ref		
Postmenopausal	336	253 (33.9)	83 (50.6)			1.630	1.012–2.628	0.045
cT stage								
T1	80	62 (8.3)	18 (11.0)	4.437	0.218			
T2	648	542 (72.7)	106 (64.6)					
T3	141	111 (14.9)	30 (18.3)					
T4	41	31 (4.2)	10 (6.1)					
cN stage								
N0	159	136 (18.2)	23 (14.0)	5.237	0.155			
N1	499	414 (55.5)	85 (51.8)					
N2	42	31 (4.2)	11 (6.7)					
N3	210	165 (22.1)	45 (27.4)					
HR status								
< 1%	361	302 (40.5)	59 (36.0)	1.141	0.285			
≥ 1%	549	444 (59.5)	105 (64.0)					
Ki‐67								
≤ 20%	91	75 (10.1)	16 (9.8)	0.013	0.908			
> 20%	819	671 (89.9)	148 (90.2)					
HER2 status								
0	188	159 (21.3)	29 (17.7)	1.180	0.554			
Low	355	287 (38.5)	68 (41.5)					
Positive	367	300 (40.2)	67 (40.9)					
cTNM stage								
II	568	482 (64.6)	86 (52.4)	8.492	0.004	Ref	Ref	
III	342	264 (35.4)	78 (47.6)			1.581	1.120–2.232	0.009
Subtype								
HR+/HER2–	312	255 (34.2)	57 (34.8)	4.395	0.222			
HR+/HER2+	227	178 (23.9)	49 (29.9)					
HR–/HER2+	138	120 (16.1)	18 (11.0)					
HR–/HER2–	233	192 (25.9)	61 (24.4)					
NAT regimens^a^								
Taxane‐based	51	39 (5.2)	12 (7.3)	1.461	0.691			
Taxane + targeted	221	180 (24.1)	41 (25.0)					
Anthracycline‐based	571	473 (63.4)	98 (59.8)					
Anthracyclines + targeted	67	54 (7.2)	13 (7.9)					

Abbreviations: HER2, human epidermal growth factor receptor 2; HR, hormone receptor; MetS, metabolic syndrome; NAT, neoadjuvant therapy.

^a^ Taxane‐based regimens including weekly paclitaxel, paclitaxel + platinum, and docetaxel + platinum; Anthracycline‐based regimens including anthracyclines + cyclophosphamide, anthracyclines (+/−cyclophosphamide) +/followed by taxanes; targeted therapy including trastuzumab +/− pertuzumab. The variables included in the multivariate analysis are age at diagnosis, menopausal status, and clinical TNM stage.

### 3.2 Association Between MetS Status and Pathological Response

**FIGURE 1:**
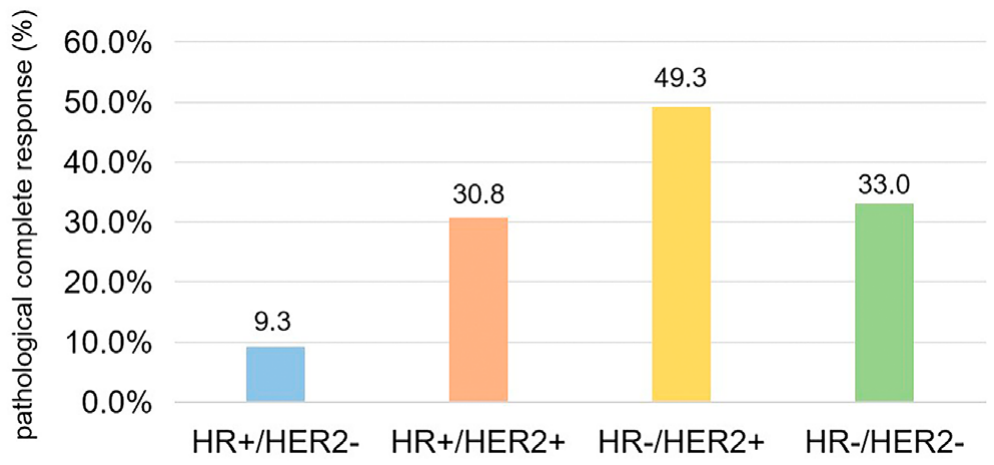
Pathological complete response rate based on the molecular subtype. HER2, human epidermal growth factor receptor 2; HR, hormone receptor.

Of the 910 patients receiving NAT, 244 (26.8%) achieved pCR. The pCR rates of luminal (HER2‐negative), luminal (HER2‐positive), HER2‐positive, and triple‐negative breast cancer were 9.3% (29/312), 30.8% (70/227), 49.3% (68/138), and 33.0% (77/233), respectively (Figure 1). Univariate analysis indicated that cT staging (*p* < 0.001), HR status (*p* < 0.001), Ki‐67 (*p* < 0.001), HER2 expression (*p* < 0.001), cTNM staging (*p* = 0.010), molecular subtype (*p* < 0.001), and MetS (*p* = 0.012) were significantly associated with the pCR rate of patients after NAT. Multivariate analysis revealed that cT staging (*p* = 0.007), Ki‐67 (*p* = 0.032), molecular subtype (*p* = 0.014), and MetS (*p* = 0.027) were the factors that were independently associated with the pCR rate. Compared with the non‐MetS group, the pCR rate was lower (OR = 0.599, 95% CI: 0.381–0.942, *p* = 0.027) in the MetS group. Moreover, the pCR rate of patients with Ki‐67 > 20% was higher than that with Ki‐67 ≤ 20% (OR = 2.213, 95% CI: 1.072–4.570, *p* = 0.032) (Table 2).

**TABLE 2:** Clinicopathological characteristics associated with pCR.

Characteristics	Total	Non‐pCR	pCR	Univariate analysis	Multivariate analysis		
		*N* (%)	*N* (%)	*p*	OR	95%CI	*p*
MetS		666 (73.2)	244 (26.8)				
No	746	533 (80.0)	213 (87.3)	0.012	Reference		
Yes	164	133 (20.0)	31 (12.7)		0.599	0.381–0.942	0.027
Age at diagnosis							
≤ 50	501	369 (55.4)	132 (54.1)	0.725			
> 50	409	297 (44.6)	112 (44.3)				
Menopausal status							
Premenopausal	574	429 (64.4)	145 (59.4)	0.167			
Postmenopausal	336	237 (35.6)	99 (40.6)				
cT stage							0.007
T1	80	46 (6.9)	34 (13.9)	< 0.001	Reference		
T2	648	468 (70.3)	180 (73.8)		0.460	0.269–0.785	0.004
T3	141	117 (17.6)	24 (9.8)		0.241	0.103–0.560	0.001
T4	41	35 (5.3)	6 (2.5)		0.295	0.093–0.939	0.039
cN stage							0.922
N0	159	114 (17.1)	45 (18.4)	0.668	Reference		
N1	499	363 (54.5)	137 (55.7)		0.911	0.585–1.421	0.683
N2	42	29 (4.4)	13 (5.3)		1.139	0.420–3.091	0.798
N3	210	160 (24.0)	50 (20.5)		0.900	0.362–2.236	0.820
HR status							
< 1%	361	220 (33.0)	141 (57.8)	< 0.001	Reference		
≥ 1%	549	446 (67.0)	103 (42.2)		0.984	0.461–2.099	0.967
Ki‐67							
≤ 20%	91	81 (12.2)	10 (4.1)	< 0.001	Reference		
> 20%	819	585 (87.8)	234 (95.9)		2.213	1.072–4.570	0.032
HER2 status							0.236
0	188	137 (20.6)	51 (20.9)	< 0.001	Reference		
Low	355	301 (45.2)	54 (22.1)		0.745	0.463–1.197	0.224
Positive	367	228 (34.2)	139 (57.0)		4.660	0.272–79.881	0.288
cTNM stage							
II	568	399 (59.9)	169 (69.3)	0.010	Reference		
III	342	267 (40.1)	75 (30.7)		0.835	0.383–1.820	0.651
Subtype							0.014
HR+/HER2–	312	283 (42.5)	29 (11.9)	< 0.001	Reference		
HR+/HER2+	227	157 (23.6)	70 (28.7)		0.729	0.043–12.496	0.827
HR–/HER2+	138	70 (10.5)	68 (27.9)		1.646	0.088–30.677	0.739
HR–/HER2–	233	156 (23.4)	77 (31.6)		4.087	1.730–9.651	0.001

*Note:* The variables included in the multivariate analysis model are MetS, cT stage, cN stage, HR status, Ki‐67, HER2 status, cTNM stage, and Subtype.

Abbreviations: HER2, human epidermal growth factor receptor 2; HR, hormone receptor; MetS, metabolic syndrome; pCR, pathological complete response.

### 3.3 Survival Analysis According to MetS Status

**FIGURE 2:**
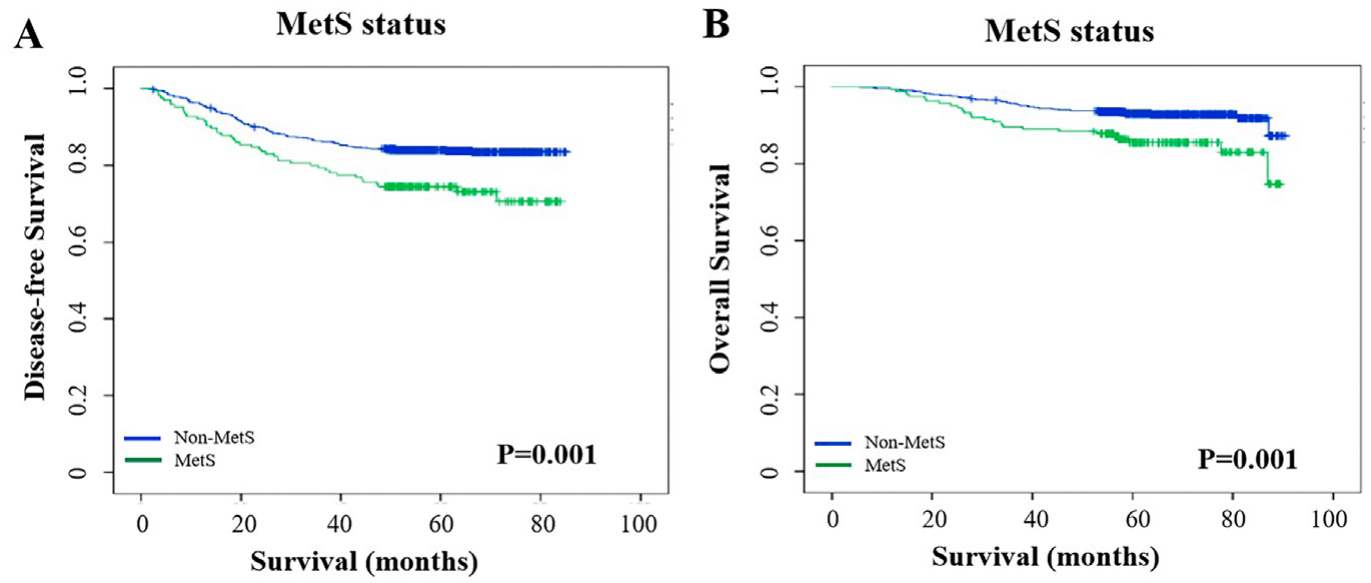
Comparison of disease‐free survival (A) and overall survival (B) between the MetS and non‐MetS groups. MetS, metabolic syndrome.

This study was followed up until December 31, 2023, with a median follow‐up time of 67.4 months (95% CI: 66.164–68.570). There were a total of 165 (18.1%) DFS events and 79 (8.7%) OS events. The overall population had a 5‐year DFS of 82.0% and a 5‐year OS of 91.7%. The 5‐year DFS of the non‐MetS and MetS groups were 83.7% and 73.1% (log‐rank test *p* = 0.001), respectively. The 5‐year OS of the non‐MetS and MetS groups were 92.8%, and 85.5% (log‐rank test *p* = 0.001), respectively (Figure 2).

The results of the univariate analysis revealed that MetS (*p* = 0.001), cT (*p* < 0.001), cN (*p* < 0.001), cTNM stage (*p* < 0.001), and pCR (*p* < 0.001) were significantly associated with DFS. The results of the multivariate analysis suggested that MetS (HR = 1.705, 95% CI: 1.201–2.421, *p* = 0.003), HR‐negative (*p* = 0.011), higher cT stage (*p* = 0.001), higher cN stage (*p* < 0.001), and non‐pCR (HR = 0.322, 95% CI: 0.195–0.529, *p* < 0.001) statuses were associated with poorer DFS (Table 3).

**TABLE 3:** Cox proportional hazards model for clinicopathological characteristics associated with DFS.

Characteristics	DFS					
	Univariate analysis			Multivariate analysis		
	HR	95% CI	*p*	HR	95% CI	*p*
MetS (vs. non‐MetS)	1.756	1.244–2.480	0.001	1.705	1.201–2.421	0.003
Age > 50 (vs. ≤ 50)	1.134	0.836–1.540	0.419			
Postmenopausal (vs. premenopausal)	1.088	0.794–1.489	0.600			
HER2 status before NAT			0.747			0.917
HER2‐low (vs. HER2‐0)	1.117	0.737–1.692	0.603	1.033	0.673–1.586	0.882
HER2‐positive (vs. HER2‐0)	0.985	0.647–1.499	0.943	1.089	0.712–1.666	0.695
HR status before NAT						
HR‐positive (vs. HR‐negative)	0.900	0.660–1.226	0.503	0.658	0.477–0.909	0.011
Ki‐67 status before NAT						
Ki‐67 > 20% (vs. ≤ 20%)	0.841	0.522–1.357	0.478			
cT stage			< 0.001			0.001
T2 (vs. T1)	1.759	0.858–3.604	0.123	1.985	0.958–4.111	0.065
T3 (vs. T1)	2.043	0.928–4.498	0.076	2.095	0.889–4.941	0.091
T4 (vs. T1)	5.766	2.523–13.175	< 0.001	5.314	2.181–12.943	< 0.001
cN stage			< 0.001			< 0.001
N1 (vs. N0)	1.870	0.986–3.549	0.055	1.958	1.016–3.774	0.045
N2 (vs. N0)	3.300	1.367–7.965	0.008	3.393	1.189–9.679	0.022
N3 (vs. N0)	7.004	3.732–13.146	< 0.001	8.243	3.365–20.194	< 0.001
cTNM stage III (vs. stage II)	3.441	2.502–4.732	< 0.001	0.855	0.433–1.686	0.650
pCR (vs. non‐pCR)	0.327	0.203–0.528	< 0.001	0.322	0.195–0.529	< 0.001
Conservative (vs. total mastectomy)	0.723	0.457–1.143	0.165			
Radiotherapy (vs. N0)	1.152	0.735–1.804	0.538			

*Note:* The variables included in the multivariate Cox regression model are MetS, HER2 status, HR status, cT stage, cN stage, cTNM stage, and pCR.

Abbreviations: DFS, disease‐free survival; HER2, human epidermal growth factor receptor 2; HR, hormone receptor; MetS, metabolic syndrome; NAT, neoadjuvant therapy; pCR, pathological complete response.

The results of the univariate analysis revealed that MetS (*p* = 0.002), the age at diagnosis (*p* = 0.015), menopausal status (*p* = 0.004), HER2 status (*p* = 0.018), cT stage (*p* = 0.001), cN stage (*p* < 0.001), cTNM stage (*p* < 0.001), and pCR (*p* = 0.039) were significantly associated with OS. The results of the multivariate analysis revealed that MetS (HR = 1.874, 95% CI: 1.149–3.055, *p* = 0.012), HER2‐0 (compared with HER2‐positive, *p* = 0.029), HR‐negative (*p* = 0.008), and higher cN stage (*p* = 0.010) were associated with poorer OS (Table 4).

**TABLE 4:** Cox proportional hazards model for clinicopathological characteristics associated with OS.

Characteristics	OS					
	Univariate analysis			Multivariate analysis		
	HR	95% CI	*p*	HR	95% CI	*p*
MetS (vs. non‐MetS)	2.139	1.330–3.440	0.002	1.874	1.149–3.055	0.012
Age > 50 (vs. ≤ 50)	1.744	1.115–2.728	0.015	1.146	0.623–2.108	0.661
Postmenopausal (vs. premenopausal)	1.910	1.228–2.970	0.004	1.502	0.819–2.755	0.189
HER2 status before NAT			0.018			0.036
HER2‐low (vs. HER2‐0)	1.056	0.616–1.810	0.844	0.966	0.545–1.712	0.905
HER2‐positive (vs. HER2‐0)	0.499	0.269–0.928	0.028	0.489	0.257–0.928	0.029
HR status before NAT						
HR‐positive (vs. HR‐negative)	0.669	0.430–1.040	0.074	0.531	0.332–0.849	0.008
Ki‐67 status before NAT						
Ki‐67 > 20% (vs. ≤ 20%)	0.880	0.439–1762	0.718			
cT stage			0.001			0.058
T2 (vs. T1)	1.480	0.532–4.112	0.452	1.858	0.657–5.256	0.243
T3 (vs. T1)	2.504	0.842–7.446	0.099	2.854	0.862–9.446	0.086
T4 (vs. T1)	5.377	1.711–16.901	0.004	4.453	1.300–15.256	0.017
cN stage			< 0.001			0.010
N1 (vs. N0)	1.600	0.614–4.168	0.336	1.720	0.640–4.622	0.282
N2 (vs. N0)	4.327	1.319–14.198	0.016	3.739	0.892–15.675	0.071
N3 (vs. N0)	6.909	2.733–17.463	< 0.001	6.243	1.749–22.292	0.005
cTNM stage III (vs. stage II)	4.329	2.664–7.035	< 0.001	1.083	0.422–2.783	0.868
pCR (vs. non‐pCR)	0.534	0.295–0.969	0.039	0.634	0.335–1.201	0.162
Conservative (vs. total mastectomy)	0.508	0.234–1104	0.087			
Radiotherapy (vs. No)	0.725	0.413–1.272	0.262			

*Note:* The variables included in the multivariate Cox regression model are MetS, the age at diagnosis, menopausal status, HER2 status, HR status, cT stage, cN stage, cTNM stage, and pCR.

Abbreviations: HER2, human epidermal growth factor receptor 2; HR, hormone receptor; MetS, metabolic syndrome; NAT, neoadjuvant therapy; OS, overall survival; pCR, pathological complete response.

**FIGURE 3:**
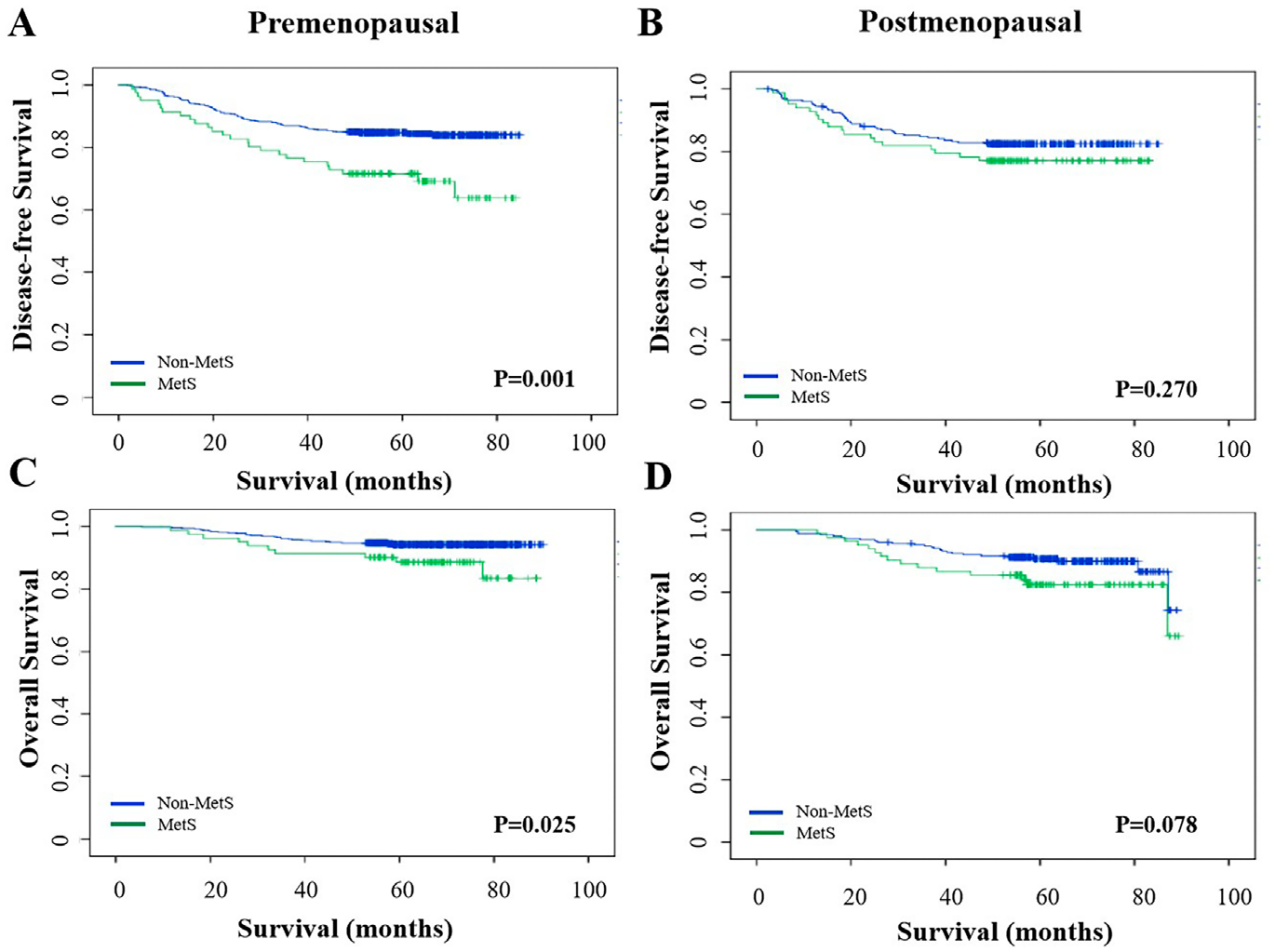
Comparison of disease‐free survival between the MetS and non‐MetS groups among the premenopausal (A) and postmenopausal (B) populations. Comparison of overall survival between the MetS and non‐MetS groups among the premenopausal (C) and postmenopausal (D) populations. MetS, metabolic syndrome.

When an exploratory subgroup analysis based on menopausal status was performed, the findings suggested that the non‐MetS group had significantly better DFS (log‐rank test *p* = 0.001) and OS (log‐rank test *p* = 0.025) than the MetS group in premenopausal women. Nonetheless, in postmenopausal women, significant differences were not observed in DFS (log‐rank test *p* = 0.270) or OS (log‐rank test *p* = 0.078) between the two groups (Figure 3). Survival analyses were conducted for patients with different molecular subtypes, and MetS was associated with poorer DFS in patients with the HR+/HER+ subtype, but not in patients with HR+/HER–, HR–/HER2+, or HR–/HER2– subtype (Figure S1). MetS was associated with OS in HR+/HER2– and HR+/HER2+ subtypes, but not in HR–/HER2+ or HR–/HER– subtype (Figure S2). The non‐MetS group was categorized into non‐MetS/DM subgroup and non‐MetS/non‐DM subgroup, and no significant difference was observed in the DFS (*p* = 0.393) and OS (*p* = 0.742) between the two subgroups. The DFS (*p* = 0.001) and OS (*p* = 0.002) of the non‐MetS/non‐DM subgroup were significantly better than those of the MetS group (Figure S3).

## 4 Discussion

Increasing evidence has shown that MetS and its components are associated with cancer risk [10, 11, 22] and poor prognosis [12]. A study observed that MetS can increase cancer mortality by 2.4‐fold [11]. Several studies have been performed on MetS and breast cancer, but few large‐sample studies are available on the impact of MetS on pCR and survival in patients with breast cancer undergoing NAT. This study investigated the association between MetS and clinicopathological characteristics, as well as the impact of MetS on the pathological response and prognosis of patients with breast cancer receiving NAT. The results implied that MetS was more common in patients with postmenopausal status or stage III and that it was associated with lower pCR rate and worse DFS and OS. However, the effect of MetS on DFS and OS was significant only in the premenopausal population, and a significant statistical difference was not observed in the postmenopausal population.

The prevalence of MetS among adults in Western countries has been documented to be 20%–25%, and the incidence rate increases significantly with age [8, 9]. In a Chinese population study, the incidence rate of MetS in patients with breast cancer receiving NAT was found to be 22.2% [23]. In this study, the incidence rate of MetS was 18.0%, which is more or less similar to the previous finding. Fluctuations in the MetS incidence rate may be caused by variations in race, living areas, diets, inclusion criteria, and the age of the study population included in the analysis in different studies. Consistent with an earlier study [6], the MetS incidence rate in postmenopausal women with breast cancer was significantly higher than that in premenopausal women in this research. Furthermore, this study identified that the incidence rate of MetS in patients with stage III was higher than that in patients with stage II. This finding could probably be attributed to the fact that abnormal blood lipid levels in MetS components were associated with higher‐stage and worse DFS [24, 25]; moreover, obesity imposed a carcinogenic effect [26, 27], and hypercholesterolemia and a high‐fat high‐cholesterol diet promoted the development of cancer [28].

In this study, a total of 244 (26.8%) patients achieved pCR after the surgery. The pCR rate was the highest in HER2‐positive breast cancer and the lowest in HR+/HER2– breast cancer, which agrees with the pCR rates of different molecular subtypes in clinical practice. Previous research findings have suggested that patients with MetS have lower pCR rates [23, 29, 30]. In this study, the outcomes of multivariate analysis alluded that cT stage, Ki‐67, molecular subtype, and MetS were independent predictors of pCR in patients with breast cancer after NAT. Achieving pCR after NAT was more difficult in patients with MetS than in those without MetS, which is consistent with previous research findings stated above. However, in this study, no association was perceived between the cN stage and pCR, which could be ascribed to the higher pCR rate of patients with HER2‐positive breast cancer after targeted treatment. This increased rate could have affected the association between the cN stage and pCR in this study.

A previous study has shown that MetS is an important risk factor for the recurrence of breast cancer [31]. MetS occurs in an environment related to adipose inflammation, which can maintain the inflammatory microenvironment and promote tumor growth [32]. Moreover, MetS components deteriorate after NAT in patients with breast cancer [33]. In this study, patients with breast cancer and MetS displayed worse DFS and OS than those without MetS. This result agrees with the findings of a study in China [23]. A large‐sample retrospective study conducted by Calip et al. [18] also observed that MetS was associated with the second breast cancer event and the increase in breast cancer‐specific mortality. Considering the poor prognosis and high staging of patients with MetS, more attention should be paid to this group. Active treatment for MetS‐related diseases must be included in the agenda for anticancer therapy. However, according to Kennard et al. [17], MetS is significantly associated with poorer DFS but not with OS. On the contrary, Buono et al. [6] reported that MetS is associated with poorer OS and BCSS in patients with early‐stage breast cancer, but not with DFS. Our analysis results of OS are inconsistent with the findings of this study, which could have been caused by the inconsistencies in the enrolled populations or the small size of the samples. More patients with stage I–II and luminal subtypes were enrolled in Buono et al.'s study than in this study. Hence, the differences might be related to variations in the clinicopathological characteristics and NAT regimens of the enrolled patients. In this study, multivariate analysis confirmed the association of both cT and cN staging with DFS and OS. pCR was associated with DFS but not OS, whereas HER2 status affected only OS. This result could be explained by the fact that the effects of pCR on DFS and OS were not analyzed in this study based on the molecular subtype. In the luminal (HER2‐negative) subtype of breast cancer, pCR and better prognosis were weakly associated, whereas in HER2‐positive and triple‐negative breast cancer, the association between pCR and better prognosis was strong [34].

The study by Bjørge et al. [19] showed that breast cancer mortality was associated with MetS in patients aged ≥ 60 years. In contrast, the exploratory subgroup analysis performed in this study revealed the presence of statistically significant differences in DFS and OS between the MetS and non‐MetS groups in premenopausal women but not in postmenopausal women. It is worth noting that in this study, premenopausal women accounted for the vast majority, whereas the number of postmenopausal women was relatively small. Moreover, in postmenopausal women, the DFS and OS of the MetS group were also numerically worse than those of the non‐MetS group. The failure to achieve a statistically significant difference could be attributed to the insufficient sample size.

In this study, compared with other investigations, a relatively large sample of patients with breast cancer receiving NAT were enrolled, which is the main advantage of this study. However, this work has certain limitations. This research was a single‐center retrospective study, which is one of its key shortcomings. Owing to the lack of data, waist circumference was not included in the analysis, and BMI was considered as a substitute for it, which constitutes another limitation. Moreover, there were differences in the treatment compliance of patients with hypertension, diabetes, and dyslipidemia in the study population, and there was a lack of treatment data, which may interfere with the results of the study. Hence, the treatments of these combined diseases were not included in the analysis, which is another shortcoming. Moreover, ER, PR, and HER2 were evaluated based on biopsy pathological reports before undergoing NAT. In addition, central laboratory validation was lacking.

## 5 Conclusion

The proportion of MetS was higher in patients with postmenopausal status or stage III. Patients with MetS had a lower pCR rate than those without MetS. In the overall population, MetS was associated with worse DFS and OS. Identical results were observed in the subgroup of premenopausal women; however, in postmenopausal women, although the DFS and OS of the MetS group were numerically lower than those of the non‐MetS group, the differences were not statistically significant. Hence, close attention should be paid to patients with MetS, and appropriate intervention measures should be implemented. Further prospective studies are needed to validate the results obtained in this study.

## Author Contributions

**Youzhao Ma:** conceptualization (lead), formal analysis (equal), methodology (lead), writing – original draft (lead). **Jingyang Zhang:** formal analysis (lead), investigation (equal), methodology (equal), software (equal). **Dechuang Jiao:** data curation (equal), validation (equal). **Xiuchun Chen:** supervision (equal), writing – review and editing (equal). **Zhenzhen Liu:** funding acquisition (lead), project administration (equal), resources (equal), writing – review and editing (equal).

## Ethics Statement

This study was conducted in accordance with the standards set out in the Declaration of Helsinki. This study was approved by the Medical Ethics Committee of Henan Cancer Hospital (Approval Number: 2022‐299). The Medical Ethics Committee of Henan Cancer Hospital granted exemption from obtaining informed consent for the study considering its retrospective nature.

## Conflicts of Interest

The authors declare no conflicts of interest.

## Supporting information


**Figure S1.** Comparison of disease‐free survival between the MetS and non‐MetS groups among the HR+/HER2− (A), HR+/HER2+ (B), HR−/HER2+ (C), and HR−/HER2− (D) populations. HER2, human epidermal growth factor receptor 2; HR, hormone receptor; MetS, metabolic syndrome.

**Figure S2.** Comparison of overall survival between the MetS and non‐MetS groups among the HR+/HER2− (A), HR+/HER2+ (B), HR−/HER2+ (C), and HR−/HER2− (D) populations. HER2, human epidermal growth factor receptor 2; HR, hormone receptor; MetS, metabolic syndrome.

**Figure S3.** Comparison of disease‐free survival and overall survival among the MetS, non‐MetS/DM, and non‐MetS/non‐DM groups. DM, diabetes mellitus; MetS, metabolic syndrome.

## Data Availability

The data presented in this study are available from the corresponding author on a reasonable request. The data are not publicly available due to ongoing studies and for patient privacy.
