# Supplementary figures and images for: Metabolic Syndrome Is Associated With Poor Prognosis in Patients With Breast Cancer Receiving Neoadjuvant Therapy
Source: Cancer Med. 2024 Dec 20;13(24):e70484. doi: 10.1002/cam4.70484 (PMC11660380; doi:10.1002/cam4.70484)

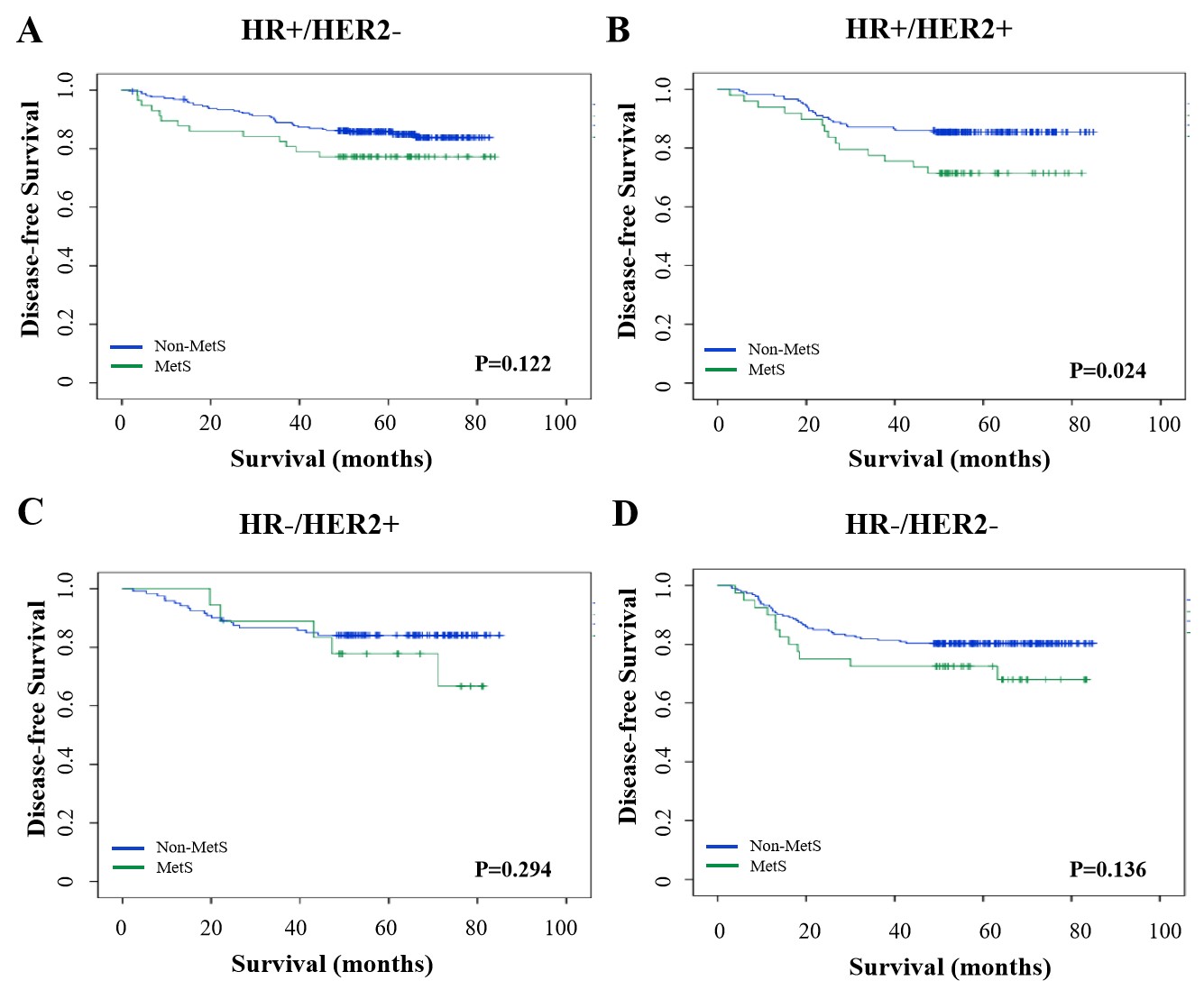

Supplement: Supplementary file 1 — Figure S1. Comparison of disease‐free survival between the MetS and non‐MetS groups among the HR+/HER2− (A), HR+/HER2+ (B), HR−/HER2+ (C), and HR−/HER2− (D) populations. HER2, human epidermal growth factor receptor 2; HR, hormone receptor; MetS, metabolic syndrome. [file CAM4-13-e70484-s002.jpg]

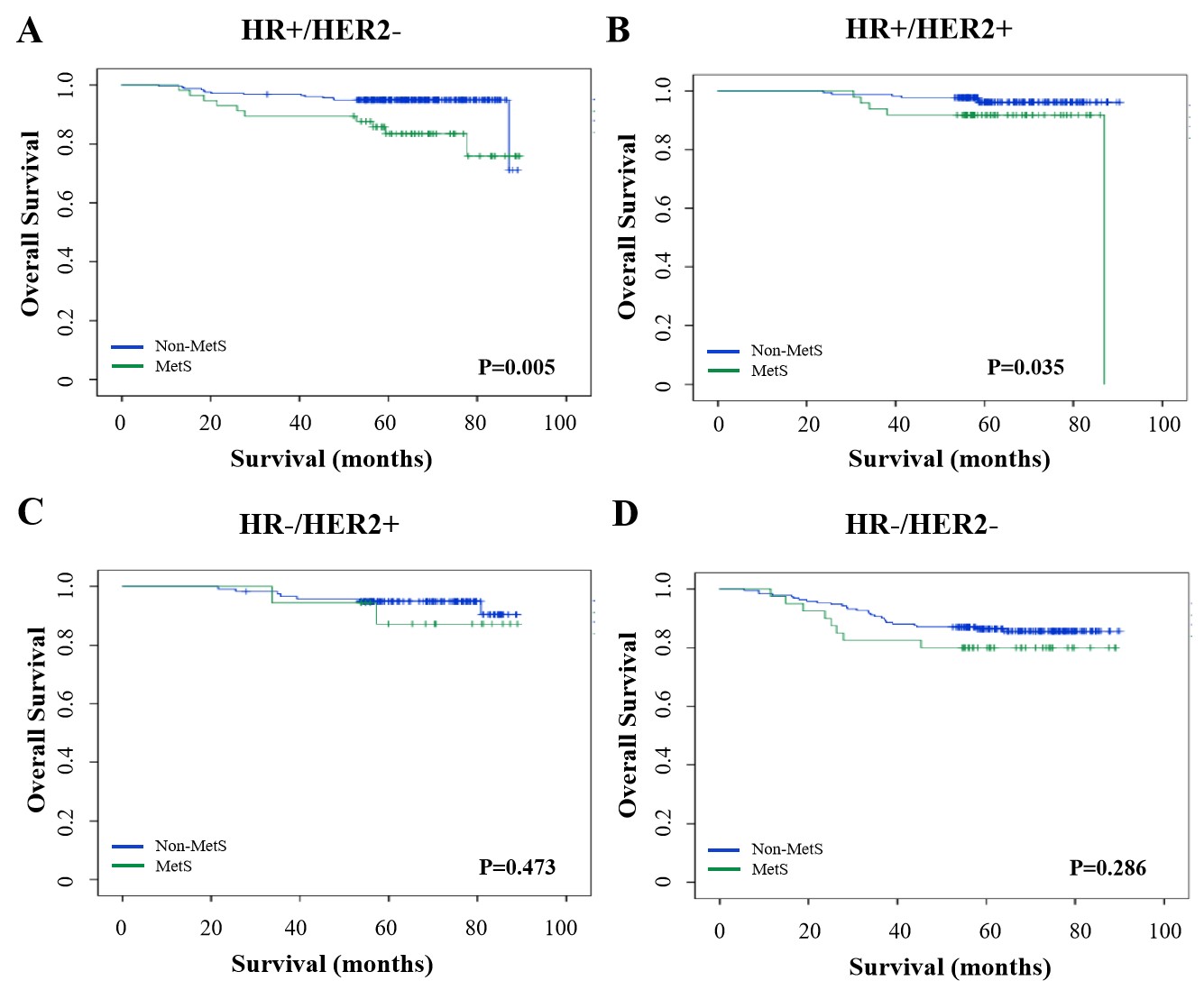

Supplement: Supplementary file 2 — Figure S2. Comparison of overall survival between the MetS and non‐MetS groups among the HR+/HER2− (A), HR+/HER2+ (B), HR−/HER2+ (C), and HR−/HER2− (D) populations. HER2, human epidermal growth factor receptor 2; HR, hormone receptor; MetS, metabolic syndrome. [file CAM4-13-e70484-s001.jpg]

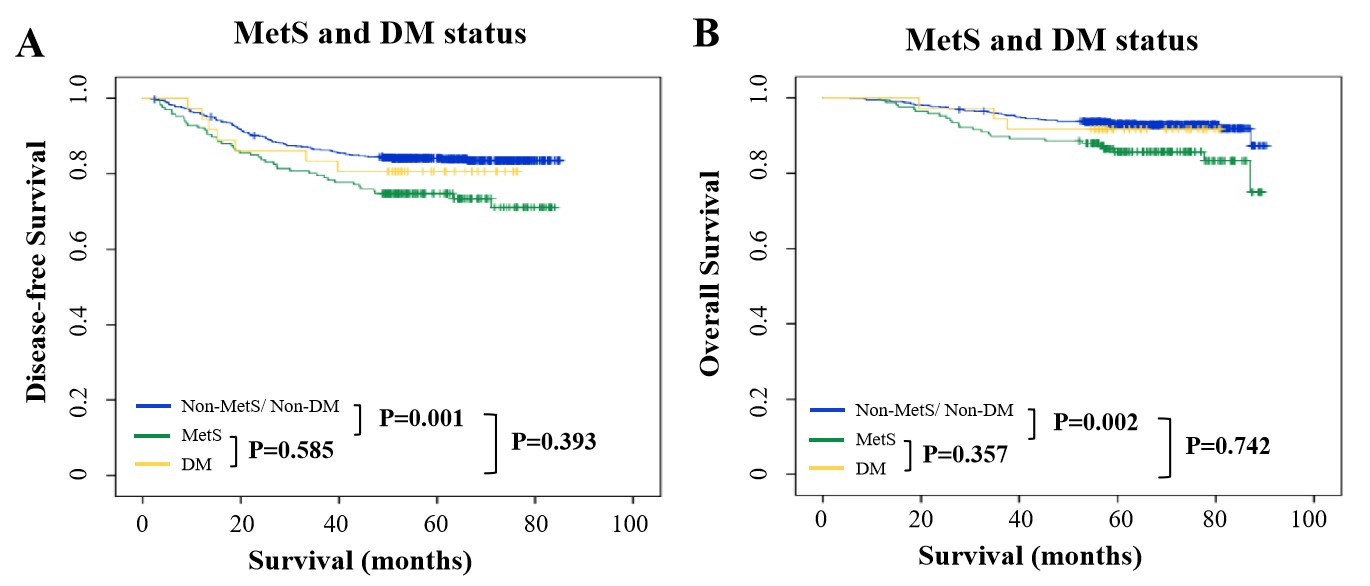

Supplement: Supplementary file 3 — Figure S3. Comparison of disease‐free survival and overall survival among the MetS, non‐MetS/DM, and non‐MetS/non‐DM groups. DM, diabetes mellitus; MetS, metabolic syndrome. [file CAM4-13-e70484-s003.jpg]
